# Editorial: Dynamics at surfaces: understanding energy dissipation and physicochemical processes at the atomic and molecular level

**DOI:** 10.3389/fchem.2024.1411748

**Published:** 2024-04-18

**Authors:** Anton Tamtögl, Helen Chadwick, Barbara A. J. Lechner, Marco Sacchi

**Affiliations:** ^1^ Institute of Experimental Physics, Graz University of Technology, Graz, Austria; ^2^ Department of Chemistry, Faculty of Science and Engineering, Swansea University, Swansea, United Kingdom; ^3^ Department of Chemistry, Functional Nanomaterials Group and Catalysis Research Center, School of Natural Sciences, Technical University of Munich, Munich, Germany; ^4^ School of Chemistry and Chemical Engineering, University of Surrey, Guildford, United Kingdom

**Keywords:** surface chemistry, catalysis, Ab initio (calculations), energy transfer, scattering spectroscopy, nanotechnology/nanomaterials, thin film growth and stability, surface diffusion

**FIGURE 1:**
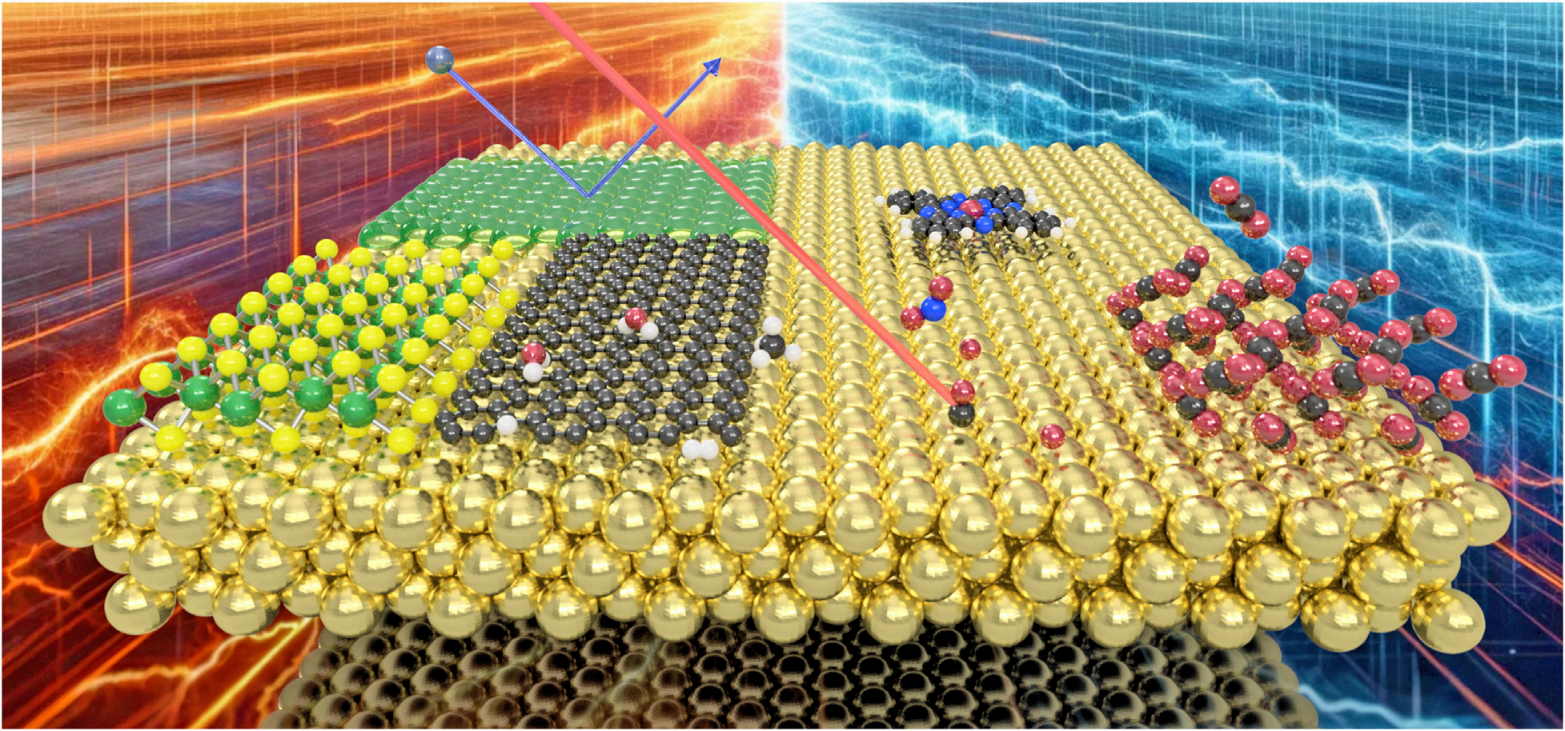
Energy dissipation processes are ubiquitous on surfaces and interfaces, from molecular motion to the formation of thin-films, determining adsorption, desorption and dissociation processes of molecules as well as the energetics upon molecular scattering from surfaces.

The processes of energy dissipation at solid interfaces (see Figure 1) are integral to numerous physical phenomena ranging from catalytic reactions and astrochemistry to lubrication and materials science including the development of nanostructures (Ertl, 2009; Yang and Wodtke, 2016; Park et al., 2019; Ollier et al., 2023). Despite its ubiquity and importance in both technological applications and natural systems these surface dynamical processes remain poorly understood (Yang and Wodtke, 2016; Park et al., 2019; Sacchi and Tamtögl, 2023; Yu et al., 2023). For advancements in fields like catalysis, electrochemistry, and photoactivated processes, a comprehensive understanding, including energy transfer from gas or liquid phase molecules to surfaces and how energy is further dissipated through various means, such as phonons and via electron-phonon coupling, is essential (Chadwick and Beck, 2017; Tamtögl et al., 2020). Over recent decades, both experimental and theoretical advancements have significantly enriched the field, enabling more detailed investigations of surface structures and surface dynamical processes (Meyer and Reuter, 2014; Nattino et al., 2016; Alducin et al., 2017; Maurer et al., 2019; Dou and Subotnik, 2020; Holst et al., 2021).

Within the current Research Topic a diverse editorial team with different approaches to the topic, including molecular and state-to-state scattering (H. Chadwick), scanning probe techniques (B. A. J. Lechner) theoretical methods (M. Sacchi) and atom/beam scattering (A. Tamtögl) collected a total of 10 original articles and one mini review including the recent progress in understanding energy dissipation in surface dynamical processes (see Figure 1 for a few illustrated examples).

The study by Sabik et al. investigates the surface dynamical motion of cobalt phthalocyanine molecules on silver using helium spin-echo (HeSE) spectroscopy, revealing that the activation energy for lateral diffusion decreases with temperature, leading to a transition from single jumps to predominantly long jumps at higher temperatures. It highlights the importance of considering a wide temperature range to capture the complete dynamics of molecular motion on surfaces.

Using the same method, Kyrkjebø et al. illustrate a stark contrast in water mobility across graphene-covered and bare Ir(111) surfaces. On graphene-covered Ir(111), water molecules exhibit significantly hindered diffusion, attributed to the trapping at specific sites within the surface’s corrugated structure. Their findings not only advance our understanding of water-surface interactions but also implicate potential impacts on the development of anti-icing and anti-corrosion materials.

Via atom-surface scattering, Maier et al. study the surface properties of 1T-TaS_2_ and TlBiTe_2_, contrasting electron-phonon coupling between these materials, and potential implications for phase transitions driven by phonons. The study of thermal expansion and interaction potentials offers valuable insights into the complex behaviour of these compounds, contributing to a broader knowledge of charge-density wave systems and topological insulators.

The scattering of keV protons through graphene is studied by Bühler et al. who challenge prior assumptions of the process. By incorporating the lattice thermal motion in simulations, they uncover that previously observed phenomena, such as the outer rainbow scattering, are artefacts of statistical averaging. At the same time, they illustrate new avenues for detailed studies of proton-graphene interactions and the orientations of graphene membranes.

Dorst et al. study the recombinative desorption of O atoms from Ag(111) by combining ion imaging techniques with temperature-programmed desorption. The hyperthermal velocity distribution of the resulting O_2_ is consistent with activated recombinative desorption but lower than state-of-the-art calculations currently predict. These results, therefore, provide a valuable benchmark for refining theoretical models of metal oxidation processes.

The influence of vibrational excitation on the sublimation of CO_2_ is investigated by Jansen and Juurlink, where they use a laser to excite the antisymmetric stretch vibration (ν_3_) of the CO_2_ impinging on the CO_2_ ice. They report that exciting ν_3_ has a negligible effect on either the sticking of CO_2_ to the ice, or the resulting structure of the CO_2_ ice despite the additional vibrational energy being greater than the CO_2_ desorption energy.

Floß et al. studied the surface-induced vibrational energy redistribution of methane scattering from Ni(111) and Au(111). Quantum state and angle-resolved measurements reveal a stark contrast in the vibrational energy conversion from ν_3_ to ν_1_ modes of methane, underlining the catalytic superiority of Ni(111) over the more inert Au(111). It thus shows a direct correlation between surface-induced vibrational energy redistribution efficiency and catalytic activity.

Tetenoire et al. elucidated the complex interplay between electrons and phonons in driving the photoinduced desorption and oxidation of CO on ruthenium surfaces. They demonstrate that phononic excitations play a pivotal role in CO desorption, while both electronic and phononic excitations significantly contribute to CO oxidation. Their research opens new avenues for optimising photochemical reactions on metal surfaces.

Xavier Jr and co-workers investigate graphene nanoribbons (GNRs) as potential catalysts for catalytic methane decomposition using density functional theory. They find that armchair edges offer lower energy barriers for hydrogen desorption, compared to zizag edges on GNRs, indicating a better regeneration potential. Highlighting GNRs as comparable to metallic catalysts for methane decomposition, their research may pave the way for sustainable hydrogen production and emphasises the significance of nanomaterials in catalytic processes for green technology.

The study by Prabhu and Groot demonstrates direct synthesis of metallic 1T Co-promoted MoS_2_ without intercalating agents via growth in a highly reducing environment. High-pressure *in-situ* reactor scanning tunnelling microscopy measurements, reveal the transformation from a disordered CoMoS_x_ phase at low temperatures to crystalline 1T slabs at around 600 K. It highlights the importance of reducing conditions in materials growth thus avoiding the need of additional chemicals.

In their mini review, Ueta et al. summarise recent studies on ortho-para conversion of hydrogen in molecular chemisorption and isolated matrix systems. These have found that nuclear-spin conversion can occur on a timescale of seconds, even for non-magnetic surfaces, and that the surface can provide a pathway for dissipating the accompanying change in rotational energy.

The collection of 11 articles within this Research Topic, though only a fraction of the extensive work in the field, highlights that understanding energy dissipation and transfer at interfaces is an extremely active area of research being studied with state-of-the-art methods both experimentally and theoretically. The importance of understanding these surface dynamical processes at the molecular level, focusing on phenomena such as photoinduced reactions, vibrational energy redistribution, and molecular diffusion on surfaces cannot be overstated. Advancements in both experimental setups and theoretical models have opened up new avenues. For example, experiments include the dynamics of larger and more complex molecules and studies of more complex surfaces compared to flat metal substrates, including two-dimensional materials and heterostructures. Similarly, enhanced computing power and the utilisation of computational clusters have enabled more sophisticated *ab initio* calculations, incorporating phenomena like non-adiabatic effects and quantum friction (Alducin et al., 2017; Chadwick and Beck, 2017; Yu et al., 2023). Furthermore, the integration of machine learning approaches promises to refine theoretical analysis further (Jiang et al., 2016; Kapil et al., 2022). Thus, the studies do not only shed light on the underlying atomic-level interactions but also pave the way for optimising materials for specific technological applications, from optoelectronics to hydrogen production.

However, challenges remain, e.g., in extending *ab initio* methods to larger systems and longer timescales and in conducting experiments under conditions that more closely mimic “real-life” parameters in catalysis to name a few (Yang and Wodtke, 2016). Moreover, while theory does well in studying specific nanosystems, there is still a need for experimental development to measure dynamical processes at tailored nanostructures or in confinement (Sacchi and Tamtögl, 2023; Yu et al., 2023). By overcoming these challenges and unravelling the mechanisms governing energy dissipation at interfaces, our community can unlock a new era of material fabrication and device control. For example, imagine designing catalysts with unparalleled efficiency, tailoring self-assembly processes for nanomaterial fabrication, or even manipulating environmental interactions on a molecular level. Future research will thus be pivotal for advancing various applications, including catalysis, energy production, and materials science by providing insights into the interaction mechanisms between molecules and surfaces, the influence of surface properties on these interactions, and the development of novel materials with enhanced functionalities.
